# The interplay of risk and protective factors for psychosocial outcome in women after induced abortion – an interview study from Germany

**DOI:** 10.1186/s12905-026-04668-9

**Published:** 2026-07-18

**Authors:** Jennifer Stünkel, Samuel Tomczyk

**Affiliations:** 1 Department Health and Prevention, Institute of Psychology, University of Greifswald, Robert-Blum-Straße 13, Greifswald, 17487 Germany; 2 University Medicine, Clinic and Polyclinic of Psychiatry and Psychotherapy, Greifswald, Germany; 3 Institute and Polyclinic for Medical Psychology and Medical Sociology, University Medical Centre, Rostock, Germany

**Keywords:** Induced abortion, Reproductive health, Interview study, Risk factor, Protective factor, Intimate partner violence

## Abstract

**Background:**

Abortions are one of the most common gynecological treatments worldwide. Although many studies investigate risk factors (RF) for a negative abortion-related psychosocial outcome (APO), less is known about protective factors (PF) and the interplay of RFs and PFs.

**Methods:**

From May to August 2022, we conducted a semi-structured interview study in Germany and interviewed 23 women (21-43 years) who had undergone an elective abortion within the last 12 months, asking them about their experiences, perceived RF, PF and APO. The data was analyzed using qualitative content analysis.

**Results:**

We found three different patterns: The first group of women found the process, especially the legal requirements, stressful but reported a mostly positive outcome with sufficient social support as PF. Women who reported neuroticism-related personality factors as RF sought less social support and more frequently used emotion-focused coping strategies such as self-care and distraction and often showed a mixed outcome. A third group of women experienced intimate partner violence (IPV) and also reported distraction as PF. Regardless, all women who survived IPV reported a negative APO.

**Conclusions:**

The majority of women find the legal requirements (e.g., mandatory counseling, waiting periods) burdensome. At the same time, they offer no benefit to women who find themselves in critical situations such as coercion and domestic violence, which was a major RF. The protection of women who survived IPV should therefore be improved and the access to abortion improved. Further research is needed to include effects on more vulnerable groups such as migrants or queer individuals.

**Supplementary Information:**

The online version contains supplementary material available at 10.1186/s12905-026-04668-9.

## Introduction

Unintended pregnancies are highly prevalent worldwide, with nearly half of all pregnancies being unplanned and approximately 30% ending in abortion [1]. In Germany, these figures are similarly substantial: one in three pregnancies is unintended, and one in twelve women undergoes an abortion during her lifetime ([2], p. 149f.). Despite this high prevalence, unintended pregnancies and abortions are often treated as discrete events, rather than as part of a complex and dynamic stress process.

Every pregnancy itself can lead to psychological stress and social challenges [3], but existing evidence suggests that women seeking abortion care are exposed to multiple, cumulative stressors that extend far beyond the pregnancy itself. These include pre-existing life strains such as financial hardship or relationship conflict, structural barriers to accessing care, procedural concerns, and post-abortion demands [4–7]. In addition, decision-making under uncertainty and the pervasive influence of stigma – both external and internalized – further intensify psychological burdens [8, 9]. Thus, abortion care must be understood not as an isolated medical intervention, but as a process embedded within a broader context of social, structural, and psychological stress.

Lazarus and Folkman’s stress-appraisal-coping framework [10] provides a useful tool to conceptualize this process. Within this model, stress is not inherent to the event itself but emerges from the interaction between situational demands and perceived coping resources. An unwanted pregnancy with a following abortion is experienced as stressful if the woman assumes that the pregnancy is a negative event (primary appraisal) and that her resources may not be sufficient to cope with it (secondary appraisal) [5, 11]. The question of resources is a particularly important one here, as according to the findings of the German ELSA study (p. 222 ff.), pregnancies are more often perceived as unwanted when the pregnant woman’s life situation is already stressful in one or more areas (e.g., relationship, financial situation) [12], meaning that her psychological resources are possibly strained already. Crucially, abortion care occupies an analytically ambiguous position within this framework: it can be interpreted as a form of problem-focused coping aimed at resolving the stressor, while simultaneously introducing additional demands that may themselves be perceived as stressful [13]. This dual role challenges simplistic narratives that frame abortion as either inherently harmful or inherently relieving.

Empirical research to date has predominantly focused on identifying risk factors associated with adverse psychological outcomes. These include prior mental health conditions, intimate partner violence, low social support, decisional conflict, and stigma [14–24]. Structural barriers further exacerbate stress, particularly among marginalized populations [4, 5]. In Germany, where abortion remains regulated under §218 StGB despite conditional decriminalization, the requirement of mandatory counseling and restricted access pathways adds an additional layer of institutional burdens [25].

However, this risk-focused perspective leaves a gap. Despite the documented challenges, most women do not experience long-term psychological harm following abortion; on the contrary, many report stable well-being and high decision satisfaction [26–28]. This apparent discrepancy points to the presence of protective factors that buffer stress and enable adaptive coping. Yet, these factors remain underexamined, particularly from the perspective of the women themselves. Existing studies provide only limited insights, suggesting roles for social support, self-efficacy, and self-esteem [29, 30], but offering little understanding of how these resources are mobilized in real-life contexts.

Moreover, the field lacks a coherent account of how risk and protective factors interact within the abortion process. Given the diversity and temporal dynamics of stressors, psychological adaptation is likely to depend on a dynamic interplay between individual, relational, and structural resources across different stages of the process. Without a more nuanced understanding of these mechanisms, only incomplete approaches can be used by prevention programs or health care providers in treating women who decide on an abortion.

The present study addresses this gap by shifting the focus from risk to resources while explicitly considering their interaction. Specifically, it asks: (a) Which protective factors (PFs) and risk factors (RFs) do the women describe spontaneously? (b) How are PFs and RFs and their interplay related to the abortion-related psychosocial outcome (APO)?

## Methods

This semi-structured interview study was approved by the ethics committee of the Universitätsmedizin Greifswald on 23rd May 2022. All supplementary material named in this manuscript can be found on OSF (https://osf.io/48edf/?view_only=dbcf2895905f432dad496da98a0c5d00). The reporting of this study follows the COREQ checklist [31] (see supp. material A1).

### Recruitment, inclusion and exclusion criteria

The study was conducted from May to August 2022 and was open to all German-speaking adults who, at the time of their participation in the study, had undergone an elective abortion in the first trimester within the last 2–12 months. This time frame was chosen to avoid biases due to immediate psychological processing of the event, since emotional fluctuations may occur in the first weeks after an abortion [32] and at the same time ensure that participants can reliably recall their experiences within the past year. The abortion must have taken place in Germany to ensure comparability of the legal and cultural processes surrounding abortion. All methods of abortion were accepted for the study in order to capture as wide a range of experiences as possible. Persons with experience of a miscarriage/stillbirth or abortions in higher trimesters in the last year due to fetal abnormalities were excluded, as were persons who had acute suicidal tendencies and/or an acute delusional phase at the time of the interview.

Participants were recruited via leaflets in gynecological practices as well as relevant groups or accounts in social networks (Telegram, Facebook, Instagram), using influencer promoting. The influencers were either organizations that provided information about abortion (e.g. Doctors for Choice e.V., https://doctorsforchoice.de/) or private persons who spoke publicly about their abortions. Recruitment followed the principle of data saturation [33]. We used iterative data collection and analysis for this purpose, starting the coding process at the same time as conducting the interviews (for further information see “Study procedure and analysis”). We considered saturation to have been achieved when no new categories could be formed from the data gathered via the interviews.

The aim was to obtain a sample that was as heterogeneous as possible, with a high age range, different relationship statuses, living in different German federal states, differences in parity, abortion methods, with the presence and absence of complications as well as a varying number of abortions. To do this, we kept a sampling plan (the sampling plan and all other supplemental material can be found at OSF). Interested participants were contacted for a telephone screening with regard to the inclusion and exclusion criteria, where they received more information about the study aims and informed consent. Suitable participants were invited for an interview.

### Definition of protective factors, risk factors and abortion-related psychosocial outcome

The aim of the study is to explore the interplay of PFs and RFs on the perceived stress and resilience of women after an elective abortion. Psychological, social or biological factors that were experienced by the women as relieving and helpful in coping with the abortion were considered PFs; RFs, on the other hand, were factors that made it more difficult for the women to cope with the abortion.

We also asked the women about their abortion-related psychosocial outcome (APO) after the abortion in order to be able to put this in relation to the PFs and RFs. A negative APO was defined as the experience of long-term negative feelings and thoughts that led to significant distress and/or impairment in the woman’s everyday life. We avoided clinical assessments so as to not pathologize the women’s experiences. This is also consistent with the broader conceptualization of psychosocial outcomes as inherently perspective-dependent constructs, which may be defined through individuals’ subjective evaluations of their own experiences [34].

A positive APO was defined as the experience of personal growth, borrowing from the idea of post-traumatic growth [35]. We do not consider abortion to be necessarily a (near) traumatic experience, but we have attempted to reflect the complex demands that women face during an abortion through this definition that may lead to personal growth after abortion [29]. We explicitly do not view a positive or negative APO as two distinct categories that represent an assessment of the participants’ state of health, but rather as a multidimensional experience of the emotional, cognitive and social consequences of the abortion. It is not uncommon for people to experience both positive and negative consequences after an abortion. If both were balanced, we referred to it as a equable APO.

### Development of the interview guide

The semi-structured interview guide was developed for this study. The aim was for the women to report freely and spontaneously on what helped them in dealing with the abortion and what burdened them. The guide (see OSF, supp. mat. A2a/b) was therefore intentionally designed in a minimalistic way and with questions that were as open as possible. The risk and protective factors were not explicitly asked, but were to be reported spontaneously by the women themselves. The most important premise here was that the participants should be regarded as “experts” for their own experiences. The questions were asked as openly and non-suggestively as possible [33]. The guide was piloted with *n* = 3 female students without abortion experience prior to the first interview and seen as understandable, ethically acceptable and feasible. While it would have been ideal to have 3 female students with abortion experience pilot the interview questions, such students were, sadly, not available.

### Study procedure and analysis

After the screening and before participating in the interview, the participants were informed separately about their rights and the anonymization process and asked for their written consent.

The interviews were conducted face-to-face at the university (*n* = 2) and online via the “Sichere Videokonferenz” video service (https://sichere-videokonferenz.de/) (*n* = 21). All interviews were conducted by the first author (female clinical psychologist, psychotherapist-in-training) as semi-structured expert interviews [33] in a one-to-one-setting. Before the recording, the participants were verbally informed a second time about their rights during and after the recording. The recording was started and ended with the knowledge and verbal consent of the participants.

The interviews were recorded with an iPod touch 6 G (without internet access) in m4a format, then transcribed and anonymized. Afterwards, all audio recordings were deleted. There was no contact between the first author and the participants before or after the study, with the exception of those women who wanted to be informed of the results.

**Fig. 1:**
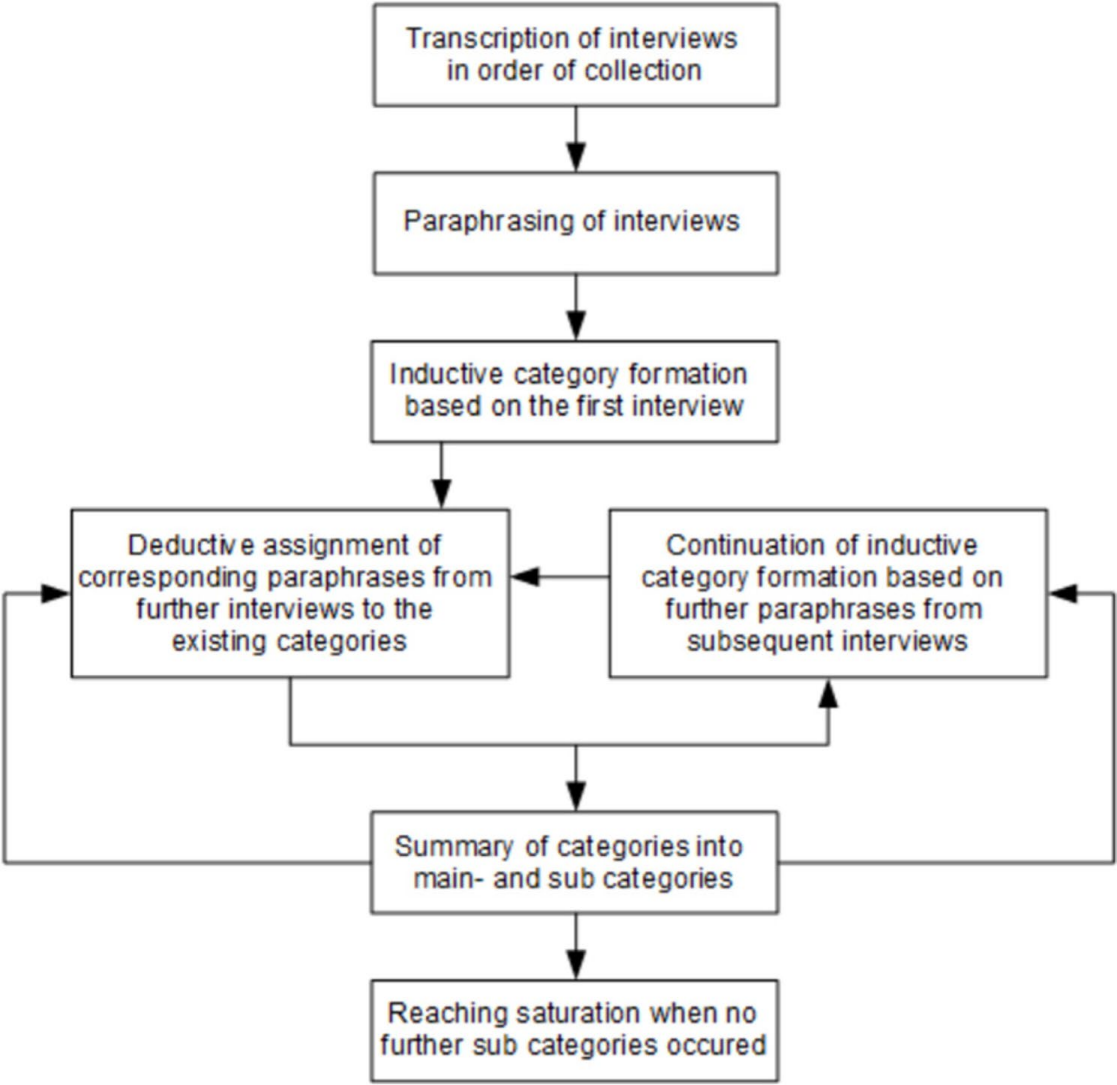
Inductive-deductive analysis process in the order of data collection

The transcripts were analyzed with MAXQDA 2.0 [36] by the first author according to qualitative content analysis in an inductive-deductive process [37]. In the first step, categories on RFs and PFs as well as positive and negative outcomes were inductively formed on the basis of the first interview. In the next step, deductive categorization of further interviews took place, while further inductive categories were formed if paraphrases could not be assigned to an existing category. This was continued until all interviews had been analyzed (cf. Fig. 1).

The categories were subsumed into head categories (e.g. reasons for abortion) and analyzed narratively. To determine the intercoder reliability, a second coding by a student (female Bachelor of Science Psychology) was carried out according to the think-aloud method [38] of two interviews (8.7% of the material) based on the final Coding Guide. The Cohen’s Kappa was κ = 0.80. This corresponds to very good interrater reliability [39].

In order to determine the APO, the paraphrases referring to the positive or negative APO of the abortion were considered. A positive, negative or neutral categorization was possible (cf. Table 1).

**Table 1:** Description of possible abortion-related psychosocial outcomes (APO)

APO	Description	Example
positive	Paraphrases describing personal growth, i.e. the participants expressed strong positive feelings (e.g. pride, gratitude) or were able to develop new perspectives in their lives in a sustainable way (e.g. more openness in sexuality, improvement of relationships).	“Abortion was positive in that regard because I had time for myself to realize some things for myself as well.”
negative	Paraphrases describing persistent psychological distress and/or negative changes in one’s life due to the abortion	“The guilt from that counseling session back then is still totally present.”
neutral	Paraphrases describing all current states related to the abortion that do not have sufficient valence to be coded as a positive or negative APO or in which the participant herself expressed unclear feelings or could not clearly state how she was currently feeling about the abortion.	“I’m fine, actually.” “It’s difficult to say how I’m doing today.”

The frequency of paraphrases with positive, negative and mixed outcomes per person was counted and participants were divided into groups with purely positive, purely negative and mixed-positive or mixed-negative outcomes for further analysis, depending on the ratio of reported positive and negative outcomes (e.g. if a participant had 8 positive outcome phrases and 2 negative, she was rated as a participant with a mixed-positive outcome; if she had just positive and no negative ones, she was rated as a participant with a purely positive outcome). If positive and negative outcomes were reported equally often, the participants were assigned an equable outcome. Using the code relation browser of MaxQDA, we looked at how many codes were coded together within a maximum distance of 10 segments. A segment was defined as the smallest unit to be analyzed that formed a meaningful unit of content [37]; each segment was assigned a code in MaxQDA. The chosen distance was applied as a pragmatic heuristic to identify thematic proximity within participants’ narratives. It was based on the observation that PFs and RFs were often discussed within close narrative proximity, particularly in descriptions of coping-related experiences following the abortion. A smaller window (e.g., five segments) proved too restrictive to capture these broader narrative contexts, whereas a larger window frequently included content that was no longer thematically related to the initially identified RF. Importantly, this co-occurrence approach does not imply causal, temporal, or functional relationships between RFs and PFs. Instead, it serves as a structured descriptive tool to explore patterns of thematic proximity within the same narrative context.

### Reflexivity statement

For our reflexivity statement, we are following the AMEE Guide No. 149 [40].

#### Personal reflexivity

The first author, psychologist and psychotherapist-in-training, was raised in Germany in a liberal family. Before the study, she was aware that the topic dealt with is a highly emotional one. Rather than adopting a clearly pro- or anti-abortion position, she approached the topic from the perspective that the ethical tensions between the interests of the pregnant person and those of the fetus cannot be fully resolved. This approach may have had an impact on the research because it created a space open to a broad range of abortion-related experiences and interpretations during the interviews.

#### Interpersonal reflexivity

The interviews took place between a young woman around 30 years as researcher and women between the ages of 20 and 48 as participants. While similarities in age and gender may have facilitated rapport in some interviews, power asymmetries between researcher and participant remain inherent to qualitative research. We sought to minimize this by taking an empathetic, approachable, and validating stance.

#### Methodological reflexivity

We decided on a constructivist approach in which the women’s experiences would be treated as their individually perceived realities. This orientation shaped both data collection and analysis by focusing on meanings, interpretations, and variations in experience, including contradictions within and across participants‘ accounts.

#### Contextual reflexivity

This study was conducted in a social and legal context in which abortion remains a controverse topic. Public debates, legal regulations, and societal attitudes toward abortion may influence both how participants describe their experiences (e.g., by shame) and how researchers engage with the topic (e.g. by reassuring an empathetic stance). Because we are aware that positive self-reports in abortion research are sometimes reinterpreted as repression or defense mechanisms, we defined the outcome categories such that a „purely positive APO“ was assigned only when exclusively positive aspects were reported.

## Results

### Sample

After initial telephone contact, twenty-four women could be screened, of whom twenty-three gave informed consent so that they could participate in the study and the interviews could be analyzed. The interviews lasted a mean of 56:06 min with a range of 27:17 and 99:33 min. The coding of the interviews resulted in a total of 4340 codes with 25 head categories (see supp. mat. A3). All participants identified as female and were on average 30.35 years old (SD: 5.76). About two-thirds of the sample was childless (see Table 2 for a descriptive statistics of the sample). One woman stated that she was Muslim, and several women expressed Christian-motivated (*n* = 5) or spiritual beliefs in the interview (*n* = 1). 52.17% of participants reported a mixed-positive (*n* = 4) or mixed-negative (*n* = 6) outcome, experiencing both distress and personal growth; purely negative (*n* = 6) or purely positive (*n* = 5) outcomes were reported about equally often. Many women reported both positive (e.g. support from partner or family) and negative experiences (e.g. the lengthiness of the process) related to abortion.

**Table 2:** Descriptive statistics of the sample

	Sample (%)
	*N* = 23
Gender	
f	23 (100.00)
Age (years)	
18–24	3 (13.04)
25–29	9 (39.13)
30–34	5 (21.74)
35–39	4 (17.39)
40–50	2 (8.70)
Parity	
0	15 (65.22)
1	1 (4.35)
2	6 (26.09)
3 or more	1 (4.35)
Relationship to Father	
yes	19 (82.61)
no	3 (13.04)
unsure^a^	1 (4.35)
Educational Status	
Lower than high school	8 (34.78)
High school diploma	15 (65.22)
Number of Abortions	
1	19 (82.61)
2 or more	4 (17.39)
Method of Abortion	
Medical	8 (34.78)
Surgical	15 (65.22)
Complications	
no	20 (86.96)
yes	3 (13.04)

^a﻿^The participant told about an “on-off-relationship” and couldn’t say if she was in a relationship with the father at that time

### Risk factors

Twenty-one women (91.30%) reported RFs in the interview in 149 paraphrases which were described as stressful before, during or after the abortion or which made it difficult to deal with the situation surrounding pregnancy and abortion. Four main categories of RFs were identified: structural/institutional, inter-individual, intra-individual and other RFs (see Table 3).

**Table 3:** Identified risk factors (RF)

	*N* women (%)	*N* Paraphrases^*^
Structural/institutional RFs	**15 (65.22)**	**55**
Stressful Experiences with Professionals	9 (39.13)	21
Stress caused by the process itself	7 (39.13)	17
Lack of Information	5 (21.74)	8
General Factors of the German Healthcare System	3 (13.04)	9
Inter-individual RFs	**15 (65.22)**	**47**
Problems with (Ex-)Partner	8 (34.78)	30
Social Stress	8 (34.78)	12
Perceived Social Stigma	2 (8.70)	5
Intra-individual RFs	**10 (43.48)**	**30**
Feelings and Assumptions	7 (30.43)	12
Trigger	4 (17.39)	11
Personality Traits	3 (13.04)	7
Other Factors	**10 (43.48)**	**15**
External Factors	5 (21.74)	8
Stressful Symptoms of Pregnancy	5 (21.74)	7

^*^referring to all responses from the entire sample

#### Structural and institutional RFs

Structural and institutional RFs include all RFs that are directly related to the decision to terminate the pregnancy and the abortion process. This category includes the sub categories „stressful experiences with professionals“, „stress caused by the process itself“, „general factors of the German healthcare system“ and „lack of information“, which often interacted dynamically in our study. Women frequently complained about the long wait time before the abortion, which they attributed either to the process itself or to a general overload of the healthcare system:

*" What really got to me was that I still walked around with it for three weeks*,* even though it was a quick abortion in my case.” (Participant 18*,* 32 years*,* purely negative outcome*,* attributed to the process itself)*^1^

*“It made things difficult for me that the abortion date [in this clinic] is only once a week.” (Participant 21*,* 31 years*,* mixed-positive outcome*,* attributed to the overload of the healthcare system)*

This was particularly stressful for women who were struggling with pregnancy symptoms:

*“I felt so terrible physically that I didn’t want to leave the house again. Waiting three days was unbearable!” (Participant 11*,* 28 years*,* mixed-negative outcome)*

There were also complaints about a lack of information regarding the abortion procedure, as either no information was available at all or it was only provided during a counseling session— yet the information given during counseling was not always perceived as impartial. Participant 11 cried as she recounted:

*“During the counseling session*,* I was told I could go to the cemetery to a burial site for stillborn babies and write a letter to my unborn child.” […] The counseling session was just so full of guilt. It was much*,* much worse than the abortion itself. My feelings of guilt were intensified by the counseling session.” (Participant 11*,* 28 years*,* mixed-negative outcome)*

The process itself was often perceived as very impersonal and hectic:

*“I think they perform so many abortions there every day*,* and so many people come in*,* that the whole process is just super streamlined… in quickly*,* get it done*,* and out again.” (Participant 19*,* 29 years*,* mixed-positive outcome)*

*“The appointment itself was*,* as you might know from comic book illustrations*,* like a cow rolling onto a conveyor belt in a factory*,* with a burger patty coming out the other end. […] That was just cold.” (Participant 8*,* 34 years*,* mixed-negative outcome)*

This process was often handled by staff who were perceived as being very insensitive. But even before and after the procedure, some women had to endure stigmatizing comments, lectures on contraception, or even a denial of medical care as these both participants stated:

*“I just told her about my nausea and how bad it is*,* and that I can’t really live my life the way I need to during that time*,* and she just said something like*,* “Yeah*,* that’s totally normal at the beginning*,* but you’re going to terminate it anyway.” […] That was like some kind of “punishment” that I wasn’t getting any medication for this nausea because*,* well*,* I’m going to terminate the pregnancy anyway.” (Participant 19*,* 29 years*,* mixed-positive outcome)*

*“It makes it difficult for me with the check-ups that my gynecologist refuses to have anything to do with the abortion.” (Participant 7*,* 30 years*,* mixed-negative outcome)*

Some women subsequently tried to switch clinics or practices, but this was in turn limited by the overburdened system, which closes the circle to long waiting time and overburdened staff:

*“But anyway*,* it’s hard enough to get an appointment with a gynecologist in general. Finding a practice that’ll take you in and then*,* um*,* yeah*,* getting an appointment on short notice is practically impossible. It’s just super hard.” (Participant 15*,* 27 years*,* purely positive outcome)*

#### Interpersonal RFs

This category included all RFs that were related to interpersonal contact in general or in the private environment (society, family, friends, partner), but not in the professional context. There were also three subcategories of interpersonal RFs: “problems with the (ex-)partner”, “social stress” (defined as “obstructive or negative aspects of social interactions”, [41], p. 215) and “perceived social stigma”.

Problems with the (ex-)partner were mentioned most frequently (30 of the 47 paraphrases) and can be categorized as abusive IPV (Partner had been psychologically and/or physically violent and/or sexually abusive in some way) or as a passive partner, overwhelmed by the situation or the abortion.

The women who had experienced IPV reported primarily sexual violence (getting raped by the partner while asleep; getting the woman drunk so the partner could have sex with her without a condom) or stalking. Participant 3 described her partner persuading her to terminate the pregnancy against her will:

*“His pressure and persuasion went on for weeks. He faked physical symptoms like hyperventilating when I said I couldn’t abort. […] I got about a hundred WhatsApp messages (from him)*^2^*during work. […] He wanted to make sure I attended all the appointments for the abortion*,* and when I didn’t reply*,* it peaked when I came out of the centre and he was standing downstairs to check if I was there.” (Participant 3*,* 43 years*,* purely negative outcome)*

Three of the five women who had experienced IPV had a purely negative outcome in terms of abortion, the other two had a mixed negative outcome. Four of the five women had already separated from or been left by the violent partner at the time of the interview.

Three participants also described the partner’s excessive demands as stressful. However, the topic could be dealt within the relationship and the relationship could be maintained. In retrospect, overcoming the situation together was seen by all three women as strengthening the partnership, in other words, as personal growth:

*“It felt as if we had reached another level of the relationship through the break-up and separation. […] There’s now more that we’ve been through together and much more understanding for each other.” (Participant 4*,* 28 years*,* mixed-positive outcome. The partner was generally described as supportive*,* but due to external circumstances such as the partner’s studies*,* the interviewee felt that the relationship was very unstable and that her feelings were not being seen by him during the abortion process)*

But also with significant others like family or friends, the support received was sometimes either lacking or felt wrong:

*„It made it harder for me that some people criticized me: Now it’s time to stop grieving! […] And*,* yeah*,* well… the fact that some people have brought that accusation against me - isn’t that enough already?“ (Participant 3*,* 43 years*,* purely negative outcome*,* lack of support)*

*„It made it harder for me when friends tried to encourage me to try.“ (Participant 14*,* 37 years*,* purely positive outcome*,* wrong support. She had the abortion done due to concerns about medical complications*,* as she had previously given birth to two children by cesarean section. Her friends tried to encourage her to carry the pregnancy to term.)*

Perceived social stigma was mentioned by two women and referred to experiences in social networks or to social expectations.

#### Intrapersonal RFs

This category includes all factors that can be located within the participants and are independent of other persons. These included “feelings and assumptions”, “triggers” and “personality traits” that the participants found stressful. The most frequently mentioned stressful feelings and assumptions were feelings of guilt about the abortion.

*„What made it difficult for me the first time I aborted was that*,* in hindsight*,* I felt I had made a real mistake.“ (Participant 23*,* 32 years*,* mixed negative outcome*,* had two abortions*,* the first one was perceived as very negative*,* due to a violent relationship*,* the cold staff and her own uncertainty*,* the second as much more empowering while being in a supportive relationship and having more positive experiences with staff)*

A second stressful factor was triggers. These included, for example, pregnancies or births among friends, but these were only mentioned as stressful by women who had either unusually stressful experiences in the context of the abortion (forced abortion through partner, severe complications, forced sex) or who had particularly wanted a child:

*“For a while after the abortion it was difficult for me to be in this flat because this is where the strong side effects happened.” (Participant 13*,* 27 years*,* mixed-negative outcome*,* experienced life-threatening side effects of the medical abortion like vomiting*,* fainting*,* seizures*,* high blood loss)*

Three women also mentioned personality traits as a RF and reported a purely negative or mixed-negative outcome. Difficulty setting boundaries was mentioned, as well as tendencies towards rumination and negative affectivity:

*„I worry a lot*,* which makes it harder for me.“ (Participant 3*,* 43 years*,* purely negative outcome)*

It is striking that women with a purely positive outcome did not report intraindividual RFs.

#### Other factors

Other factors include stressful symptoms of pregnancy (e.g. nausea, hormonal fluctuations) and external factors (e.g. stressful work situation, lockdown restrictions in the wake of the COVID pandemic or being overwhelmed with the parallel care of children in the household). Both pregnancy symptoms and other external factors were experienced as stressful by women of all outcomes.

### Protective factors

Twenty-two women (95.65%) reported PFs in 220 paraphrases that were mentioned as helpful before, during or after the abortion or that made it easier to process the situation surrounding pregnancy and abortion. Nine different PFs were mentioned in the interviews (see Table 4).

**Table 4:** Identified protective factors (PF)

	*N* (%)	*N* Paraphrases*
Perceived Social Support	**20 (86.96)**	**120**
… from Private Environment	20 (86.96)	95
… from Professionals	8 (34.78)	25
Self-Care	**17 (73.91)**	**60**
Withdrawal/Time for Oneself	8 (34.78)	15
Active Coping	7 (30.43)	14
Distraction	6 (26.09)	17
Grieving/Farewell	5 (21.74)	9
Pain Management	3 (13.04)	5
Cognitive Factors	**9 (39.13)**	**16**
Accepting Methods	4 (17.39)	6
Rationalising Methods	4 (17.39)	5
Imaginative Methods	3 (13.04)	5
External Factors	**9 (39.13)**	**20**
Factors in the Work/Education Environment	4 (17.39)	5
Elimination of Risk Factors	3 (13.04)	7
Passing of Time	3 (13.04)	5
Own Children	2 (8.70)	3
Ending Pregnancy early	1 (4.53)	2
New Start after Abortion	1 (4.53)	1

^*^referring to all responses from the entire sample

#### Perceived social support

Perceived social support was named as a central PF by almost all women, regardless of the APOs. Perceived social support can be defined as “the subjective conviction of receiving support from the social network when needed, or the assessment of being able to fall back on resources of the social environment” ([41] p. 213). Emotional support describes the provision of empathy, trust and care by the environment; instrumental support is to receive practical help with everyday problems, e.g. borrowing something or being relieved of duties [41]. The women mainly experienced social support from their private environment (friends, partners, family) as a PF, especially emotional support through conversations:

*“A friend just listened to me*,* just asked me how I was doing. Without much advice or anything. That was good. I could tell him everything and he totally took his time.” (Participant 4*,* 28 years*,* mixed-positive outcome)*

In three cases, the women also reported social practical support as helpful, e.g. through transportation to the clinic or in organizing everyday life during the abortion.

At the same time, however, some women also reported that they had sometimes held back and not spoken about the abortion out of concern about stigma or consideration for others, such as people hoping to have children:

*“I can’t bring myself to do that to my sister (tell her about the abortion) because I know just how much she has suffered from her unfulfilled desire to have children.” (Participant 5*,* 36 years*,* purely positive outcome)*

*“Yes*,* I’m worried I might end up with someone who isn’t sensitive enough when it comes to this topic. [… ] I make myself very vulnerable when I open up.” (Participant 8*,* 34 years*,* mixed-negative outcome)*

Without exception, all women also had positive experiences of social support from professionals during the abortion process, when they were experienced as understanding, empathetic, supportive and giving space and provided information on the further process after the abortion, e.g.:

*“It was helpful that in the post-abortion check-up there was a very nice*,* older gynecologist who took an extremely long time.” (Participant 4*,* 28 years*,* mixed-positive outcome; was afraid that she would no longer be able to get pregnant)*

Participant 3, who suffered from pressure from her partner to abort, reported that, although she felt well cared for by the staff, she was unable to confide in them because of the domestic violence:

*“His pressure and persuasion went on for weeks. […] So I lied to everyone at the counseling center. […] I lied because I thought that if I told them my boyfriend was forcing me*,* I wouldn’t get the counseling voucher [which is necessary for abortion in Germany*,* author note].”(Participant 3*,* 43 years*,* purely negative outcome)*

#### Self-care

Self-care comprises all PFs in which the participants independently cared for their well-being after the abortion, including the sub categories „withdrawal/time for oneself“, „active coping“ with the abortion (e.g. working through it with the partner or own confrontation), „distraction“ (e.g. through hobbies or daily routine) and „pain management“ (e.g. painkillers, hot water bottle). Some women found it helpful to say goodbye to the fetus and grieve, especially if the fetus was already regarded as their own child:

*“It was just my child. Whether it was just tissue or whatever was actually there… but to me*,* it’s just … a part of me … That’s my child. […] I made myself a little personal shrine with ultrasound photos in a frame that’s blue and pink*,* because I don’t know if it’s a girl or a boy. (Participant 12*,* 24 years*,* equable APO)*

*I said [to the gynecologist]: “I want to see my baby*,* let me see it!” I do want to terminate the pregnancy*,* but I still want to see it one last time! […] I’ve made myself a little corner at home*,* and that’s good enough for now.” (Participant 9*,* 37 years*,* purely negative outcome)*.

#### Cognitive factors

Cognitive PFs were thoughts, attitudes and assumptions that were experienced as helpful in processing the abortion. A distinction could be made between accepting (e.g. radical acceptance), rationalizing (e.g. argumentative affirmation of the decision) and imaginative approaches (e.g. imagining the child as a little soul).

*„It helped me to simply check the box and say: it’s done now*,* it is what it is.“ (Participant 23*,* 32 years*,* mixed-negative APO*,* accepting)*

*„It helped me to think this through sensibly and realistically.“ (Participant 9*,* 37 years*,* purely negative APO*,* rationalizing*,* with 5 children and no partner*,* recognizing that she can not afford the time and money to raise a sixth child)*

*„It helped me to imagine that this was a little soul that had come to me and that was now moving on or would come to me again at some other time. That helped me a lot.“ (Participant 1*,* 22 years*,* equable APO*,* imaginative)*

#### External factors

External factors were understood as factors that were not directly related to the abortion and over which the women had no significant influence. These included factors in the private environment, mainly their own children and positive previous experiences with other close relatives with abortions; factors in the work/education environment, e.g. job security, flexible working hours and the possibility of temporarily reducing their own workload (postponing exams, being able to take sick leave), as well as the elimination of RFs, mainly the physical distance to abusive partners and the reduction of pregnancy symptoms after the abortion, but also the passing of time.

#### Other factors

Two women reported other PFs that could not be sorted into these four categories: “a new start after abortion” and “ending the pregnancy early” to avoid seeing a heartbeat:

*“It actually helped me that the pregnancy was terminated at a very early stage – in the sixth week of pregnancy. […] I was actually relieved that there was no heartbeat yet.” (Participant 14*,* 37 years*,* purely positive outcome*,* who wished for a third child but decided against it out of fear of health risks for herself)*

### Interplay of risk factors, protective factors and abortion-related psychosocial outcome

On average, the women reported 3.3 PFs and 3.0 RFs (Table 5). There is a mentionable group difference in the total number of RFs: women with purely positive APOs reported fewer RFs (m = 1.8) than women with other APOs, who reported 3-3.5 RFs. In the number of paraphrases, an increase can be seen with a worse APO, too. On average, women with a purely positive APO reported their RFs in 2.6 paraphrases, whereas women with a purely negative APO reported them almost four times as often (10.7 paraphrases), which is mainly due to the women who reported IPV.

**Table 5:** PFs and RFs and No. of paraphrases for PFs and RFs, sorted by APO groups

APO (*n*)	No. of PF (mean)	No. of RF (mean)	Paraphrases PF (mean)	Paraphrases RF (mean)
Purely positive (5)	3.8	1.8	9.2	2.6
Mixed positve (4)	3.8	3.5	10.0	6.8
Equable (2)	3.5	3.5	6.5	6.0
Mixed negative (6)	3.3	3.2	10.5	5.5
Purely negative (6)	3.3	3.0	9.7	10.7

Overall, it can be observed that women with a purely positive APO not only reported fewer RFs, but also in fewer paraphrases. Although women with purely negative APOs reported on average about as many RFs as the other APO groups.

On the other hand, there were hardly any differences in the number of protective factors (m = 3.5, range: 3.3–3.8) or paraphrases (m = 9.57, range: 6.5–10.5) between the groups.

Nevertheless, differences between experienced RFs and utilized PFs became apparent when considering the APO, particularly when examining how specific RF-PF constellations cluster within a maximum distance of 10 segments. Due to their size, the corresponding tables can be found on OSF (A4, A5).

With regard to structural and institutional RFs, women across all outcome groups reported negative experiences; women who generally reported stressful professional interactions more often also perceived the procedure and broader healthcare system factors as burdensome. However, these were described in greater detail and frequency by women with purely negative or mixed-negative APOs. These RFs frequently co-occurred with the use of social support and self-care as PFs. Women who experienced stressful interactions with professionals or perceived the abortion process itself as burdensome primarily sought support within their private environment – most often from friends, less frequently from partners, and least frequently from family. On the other hand, professional support was perceived as particularly helpful by women with purely negative or mixed-negative APOs. In addition, structural and institutional RFs were often reported together with self-care strategies, especially in the sense of actively coping with the experience.

Interpersonal RFs were reported across all outcome groups, whereas IPV was observed primarily among women with negative APOs. In these cases, references to abortion-related triggers frequently appeared in close proximity to IPV. Compared with other women, those reporting IPV provided fewer references to social support, particularly partner-based support. IPV was also frequently linked to self-care practices, especially distraction through hobbies or everyday routines. Among all RFs, IPV most often appeared together with references to distraction. References to professional support were likewise comparatively frequent among women reporting IPV.

A notable distinction emerged regarding intraindividual RFs. Women with a purely positive APO did not report such factors, whereas women with purely or mixed-negative APOs referred to them substantially more often than those with equable or mixed-positive APOs. Intraindividual RFs – particularly personality traits and negative assumptions or cognitions – frequently co-occurred with social stress, especially insufficient social support, and showed distinct patterns of co-occurrence with PFs: References to personality traits as RFs most frequently appeared in proximity to withdrawal as a self-care strategy, whereas negative assumptions or cognitions more often co-occurred with distraction. At the same time, references to social support were comparatively infrequent in passages involving negative assumptions or cognitions and were limited to occasional mentions of friends, while references to support from partners, family members, or professionals were absent. In contrast, reports of negative feelings more frequently appeared alongside references to social support from the private environment and less often in proximity to withdrawal or distraction. Overall, intraindividual RFs frequently co-occurred with self-care strategies.

Across PFs more broadly, social support differed across APO groups APO: women with a purely positive APO reported the highest levels of social support, while those with a purely negative APO reported the lowest. Self-care practices, in contrast, appeared largely independent of APO; the only exception was grieving and farewell processes, which were reported exclusively by women with predominantly negative APOs. Finally, cognitive PFs were mentioned more frequently as APO became more negative.

## Discussion

In this paper, we present the results of 23 semi-structured interviews. Due to the secondary appraisal of Lazarus and Folkman’s stress-appraisal-coping theory [10]. The aim was to learn about key PFs and RFs during secondary appraisal from the women themselves and to examine whether there are any notable connections between certain PFs and RFs and the women’s APOs, which follows a subjective, participant-centered approach, consistent with the view that psychosocial outcomes are defined by individuals’ lived experiences rather than exclusively by clinically derived criteria [34].

When the results are combined, three types of RF–PF constellations emerge. The first type of woman is characterized by reports of structural and institutional stressors, such as burdens associated with the abortion process or stressful interactions with professionals, alongside comparatively high levels of social support. Women in our study whose accounts reflected this type were more often represented in the positive or mixed-positive APO groups. In addition, these groups reported fewer RFs overall than women with more negative APOs.

Several interpretations may account for this pattern. One possibility is that women represented by this type experienced fewer or less severe RFs overall. In the present sample, for example, IPV was not reported by any woman with a purely positive APO. Another possibility is that abortion-related circumstances were appraised differently. Research suggests that more positive affect may be associated with less negative appraisals of everyday stressors [42]. At the same time, women experiencing unintended pregnancies are more likely to report stress or dissatisfaction in other areas of life [12, 43]. The prominence of social support within this type may indicate that supportive interpersonal relationships constitute an important resource within abortion-related experiences, although the present data do not permit conclusions regarding causal mechanisms.

This type appears broadly consistent with findings from large longitudinal studies such as the German ELSA Study [12], the American Turnaway Study [21, 28, 44], and the Dutch DAMHS Study [45]. These studies suggest that abortion-related distress is often temporary and generally declines over time without resulting in long-term mental health problems. Thus, the first type may reflect a comparatively adaptive pattern of abortion-related adjustment characterized by the co-occurrence of stressors and substantial social support.

The second type is characterized by reports of partner pressure and intimate partner violence (IPV). In the present sample, women whose accounts reflected this type were more often represented in the negative APO groups. Compared with other participants, they provided fewer references to social support as a PF and more frequently described distraction as a form of self-care. Previous studies like the ELSA study reported that women who experience IPV are less likely to be socially integrated ([12], p. 377 ff., [46]), which may also be a result of manipulation by [47] or isolation through violent partners ([12, 48] and has more than twice the risk of developing PTSD [49]. Taken together, these findings may suggest that IPV represents a particularly burdensome context within which abortion-related experiences are embedded. However, the present data do not allow conclusions regarding the extent to which IPV itself contributes to APO or how individual RFs and PFs interact over time. An alternative interpretation is that the PFs observed within this type, such as professional support or self-care strategies, may have mitigated some of the psychosocial burden and potentially contributed to outcomes that were less negative than might otherwise have occurred. Because the study design does not permit counterfactual comparisons, this possibility remains speculative. The predominance of negative APOs within this type nevertheless aligns with broader evidence showing that IPV is associated with a range of adverse psychosocial outcomes and increases the likelihood of unintended pregnancy [50, 51]. Estimates further suggest that approximately one in six to seven women seeking abortion services experience IPV [12, 52]. These findings underscore the importance of ensuring that abortion care settings are equipped to identify and support women affected by IPV.

The situation is more diffuse for the third group. These women typically report intra-individual RFs such as personality traits or negative assumptions/cognitions, which may reflect characteristics that overlap with aspects of neuroticism [53, 54]. They cite social support less often as an PF, but sometimes cite too little support as an RF. Instead, they describe withdrawal or distraction more often as PFs. It has been widely documented across different ethnicities, genders, ages and occupational groups that neuroticism is associated with avoidance- and emotion-focused coping [55–59] and lower perceived social support [60]. Pregnant women with high neuroticism tend to emotion-focused coping, too [61]. Neuroticism is also a predictor for PTSD symptoms after abortion [16]. Women whose accounts reflected this type were not represented in the purely positive APO group, although their APOs were not uniformly negative.

What is particularly striking about the current state of research is that stigmatization played a relatively minor role in our study. According to the ELSA study, more than half of all women conceal their abortion from some of those around them out of fear of stigmatization [5]. At the same time, (anticipated) stigmatization is one of the most relevant RFs for a negative APO after abortion in international studies [23, 24]. In our study, however, stigmatization from the surrounding community is not reported at all, but it is more commonly reported by healthcare professionals. Only two women reported stigmatization, mainly referring to anonymous opinions on the internet. It is possible that there is a sampling bias here, and that only women who were not afraid of stigmatization – or who did not experience it – came forward. On the contrary, the women in our sample generally seemed to know very well whom they could confide in and whom they could not. The fear of stigma does not necessarily keep women from opening up, as many women still do so despise stigma [62, 63] or have been forced to do so because they, for example, need practical support with the abortion [64]. Weighing up whether to confide in someone – and, if so, whom – can be part of the perceived stigma [62]. The women in our study also repeatedly considered whom they wanted to confide in and whom they did not.

### Limitations

This study has several limitations: Methodologically, the operationalization of the APO used here has its limitations. The APO was determined based on the relationship between positive and negative outcome paraphrases. Although frequency analyses represent a practical, transparent, and well-established method in qualitative research, as they enable the systematic condensation of extensive text data and yield intersubjectively verifiable results [65], there is a risk of losing depth of content, since individual outcome paraphrases are weighted equally regardless of their subjective significance [66]. Accordingly, individually reported consequences – regardless of their number – can have varying degrees of impact on the lives of the participants.

It is also important to consider, in the context of distressing or potentially traumatic experiences, that negative content may be reported less frequently or in less detail. Avoidance, fragmentation, or limited verbalization can be central features of the processing of distressing experiences [67]. These phenomena can lead to negative consequences being underrepresented in the interview context, without this reflecting their actual subjective relevance.

Furthermore, the influence of social desirability cannot be excluded. Respondents may tend to frame their accounts in a way that appears socially acceptable or meets certain implicit expectations. This can lead to positive aspects being emphasized more strongly or negative aspects being downplayed [68]. In the present context, for example, it is conceivable that positive outcomes are emphasized so as not to unsettle or deter other women.

Moreover, the criterion for a positive outcome — defined in terms of personal development— is comparatively stringent. It does not merely capture the absence of psychological distress or symptoms but requires a reported increase in personal growth. However, theoretical frameworks such as Posttraumatic Growth suggest that such developmental processes are more likely to occur in the context of having experienced and successfully coped with a significant stressor [69]. This has both theoretical and practical implications for the interpretation of the present findings. From a theoretical perspective, it is open to question whether a positive outcome following an abortion should only be assumed when personal growth is reported. Such a narrow definition risks implicitly over-emotionalizing the experience of abortion.

From a practical standpoint, this operationalization may lead to biased classifications. For instance, a woman who did not experience the abortion as highly distressing and therefore did not engage in an in-depth emotional processing may not report personal growth – despite also not experiencing negative consequences. Within the present framework, she would not be classified as having a positive APO. Paradoxically, her outcome could thus be interpreted less favorably than that of a woman with a mixed-positive APO, who reports both positive and negative consequences. This highlights that the criterion of personal development may not adequately capture all forms of adaptive coping. In the present sample, two cases with a neutral outcome may reflect this pattern. Member checking [70] would have been one way to validate the results, particularly the APO, but it was not possible due to time constraints. Importantly, all co-occurrence findings reported in this study reflect thematic proximity within a predefined coding window and should be interpreted as descriptive patterns within participants’ narratives rather than as evidence of causal or functional relationships between risk and protective factors.

With regard to the sample, selection bias plays a role. Usually, highly educated and younger people tend to participate in studies more frequently [71]. This is exacerbated by a technical bias: people who do not have access to the internet were excluded from the study, which often applies to older and less educated people [72]. And although we tried to invite them by contacting group-relevant influencers, people of color, with migration background and queer people are missing in our sample. Hence issues of intersectionality could not be explored, although the multiple stigmatization caused by abortion and intersectionality urgently needs research [73]. Since it is known that these groups report more frequent experiences of stigmatization and discrimination [74], it is possible that RFs are added or have a more serious influence or that the PFs mentioned here are not sufficient. However, it should be noted that, according to the World Value Survey, Germany is a very liberal country. In countries with higher levels of stigmatization, social support may be much less available as a PF and may force women to seek other coping strategies. According to our results, in which social support cannot be balanced by any other PF, this could lead to poorer psychosocial outcomes for women.

Sampling bias may also have played a role, as evidenced by the fact that only a relatively small number of women reported experiencing stigmatization.

Nevertheless, we achieved a very heterogeneous sample in our study, with a wide age range, varying parity, educational status, relationship status, religious background, number of abortions with and without complications, and APOs, so that the results can be assumed to be highly transferable.

To the authors’ knowledge, this is the second study worldwide to explicitly survey PFs after an induced abortion (in addition to Barraza Illanes & Calvo-Francés [29]) and the first to ask about them in a semi-structured interview with directly affected persons. We have declined to provide a clinical assessment, as we believe that women are best able to assess their own mental state. This provides the opportunity to accurately depict subclinical phenomena without pathologizing the feelings of the person with abortion experience.

### Implications for research and practice

Our findings suggest that abortion-related experiences are best understood in terms of recurring RF-PF constellations rather than isolated risk or protective factors. Across the sample, different configurations of structural, interpersonal, and intraindividual RFs co-occurred with distinct patterns of perceived support and APOs, indicating that contextual combinations may be more informative than single-variable associations.

From a research perspective, this implies that future studies may benefit from focusing on such constellations and their interrelations. In particular, the present typology suggests that RFs and PFs tend to cluster in meaningful patterns (e.g., structural stressors alongside social support, IPV alongside limited social support, and intraindividual RFs alongside reduced perceived support and increased self-care). This constellation-based approach can help to better understand the heterogeneity of abortion-related experiences. In addition, longitudinal designs would be needed to examine how stable or context-dependent these patterns are over time, and whether they reflect enduring individual differences, situational responses, or dynamic interactions between both.

A further implication concerns the prominent role of intimate partner violence (IPV) as a key contextual factor. IPV was embedded in a broader pattern characterized by reduced perceived social support, increased triggers, and higher representation in negative APO groups. This suggests that abortion care contexts may benefit from closer integration with services addressing interpersonal violence, as well as from systematic attention to safety and disclosure conditions within clinical and counseling settings. Women affected by IPV apparently cannot always open up during counseling sessions, so alternative ways must be found to reach these women.

From a practice perspective, these findings may support approaches that complement professional counseling with low-threshold, context-sensitive forms of support. Peer-based accompaniment models, such as voluntary „buddy“ programs, may represent one such approach by providing additional relational continuity alongside formal care. Some buddy programs already exist in Germany on a voluntary basis, in which volunteers accompany women to all appointments and also offer support after the abortion [75]. There are some qualitative results for IPV peer support programs (for a scoping review see [76]), but none for abortion buddy programs. We see this as a promising field of future research.

Overall, the results suggest that abortion care and counseling may benefit from a differentiated perspective that takes into account the diversity of RF–PF constellations and their varying implications for perceived support and psychosocial outcomes. At the same time, the exploratory nature of the study does not allow conclusions about causal pathways, and further research is needed to clarify how these patterns develop and interact over time.

## Supplementary Information

Supplementary Material 1.

Supplementary Material 2.

Supplementary Material 3.

Supplementary Material 4.

Supplementary Material 5.

Supplementary Material 6.

## Data Availability

The data that support the findings of this study are available from the corresponding author upon reasonable request.

## Footnotes

1 The interviews were conducted in German. All translations of the quotes are by the first author.

2 Notes in paranthesis are added for clarity by the first author.

## Abbreviations

APO: Abortion-Related Psychosocial Outcome
IPV: Intimate Partnership Violence
PF: Protective Factor
RF: Risk Factor
