# Supplementary material for: The interplay of risk and protective factors for psychosocial outcome in women after induced abortion – an interview study from Germany
Source: BMC Womens Health. 2026 Jul 18;26:365. doi: 10.1186/s12905-026-04668-9 (PMC13380845; doi:10.1186/s12905-026-04668-9)
Supplement: Supplementary file 2 — Supplementary Material 2. [file 12905_2026_4668_MOESM2_ESM.pdf]

Leitfaden zum halbstrukturierten Interview zu Schutz- und Risikofaktoren nach Schwangerschaftsabbruch

1. Aufklärung

- Vorstellung, Aufklärung über Schweigepflicht, Datenschutz, Sinn und Zweck der Studie, über die Aufnahme und den Zeitumfang und die Freiwilligkeit der Beantwortung der Fragen
- Klären von Fragen seitens der Probandin vorab
- Erheben des Codes:
  - 1. Die beiden letzten Buchstaben des Geburtsnamens Ihrer Mutter
  - 2. Die Anzahl der Buchstaben des (ersten) Vornamens Ihrer Mutter
  - 3. Die beiden letzten Buchstaben des (ersten) Vornamens Ihres Vaters
  - 4. Ihr eigener Geburtstag (nur der Tag, nicht Monat und/oder Jahr).

2. Erleben der Schwangerschaft

- Wie ging es Ihnen, als Sie von Ihrer Schwangerschaft erfahren haben?
- In welchen Lebensumständen befanden Sie sich gerade, als Sie von der Schwangerschaft erfahren haben?
- Wie haben Sie den Prozess der Entscheidungsfindung erlebt?

3. Erleben des Schwangerschaftsabbruchs

- Wie haben Sie den Schwangerschaftsabbruch für sich ganz persönlich erlebt?
- Gab es etwas, das Ihnen geholfen hat, dieses Ereignis zu verarbeiten? (Schutzfaktoren) Wenn ja, warum?
- Gab es etwas, das es Ihnen erschwert hat, dieses Ereignis zu verarbeiten? (Risikofaktoren) Wenn ja, warum?
- Gibt es etwas, was Sie sich gewünscht hätten, um den Eingriff (noch) besser verarbeiten zu können? Wenn ja, was und warum?

4. Konsequenzen

- Wie geht es Ihnen heute in Hinblick auf den Schwangerschaftsabbruch? (Pos/neg Outcome)
- Was für Gedanken und Gefühle haben Sie nun, wenn Sie daran zurückdenken, was Sie alles erlebt haben im Zusammenhang mit dem Schwangerschaftsabbruch? (Pos/neg Outcome)
- Spielt das Thema bei Ihnen im Altag noch eine Rolle? Wenn ja, inwiefern? (Pos/neg Outcome)

5. Abschluss des Gesprächs

- Gibt es noch etwas Wichtiges zu dem Thema, was ich vergessen habe zu fragen?
- Klären des emotionalen Zustands der Probandin, ggf. Nachbesprechung und nochmaliger Hinweis auf das Informationsblatt mit den Beratungsangeboten
- Bedanken und verabschieden von der Probandin
