# Supplementary material for: The interplay of risk and protective factors for psychosocial outcome in women after induced abortion – an interview study from Germany
Source: BMC Womens Health. 2026 Jul 18;26:365. doi: 10.1186/s12905-026-04668-9 (PMC13380845; doi:10.1186/s12905-026-04668-9)
Supplement: Supplementary file 3 — Supplementary Material 3. [file 12905_2026_4668_MOESM3_ESM.docx]

# Guideline for semi-structured interviews on protective and risk factors following abortion

## 1. Briefing

• Introduction, information about confidentiality, data protection, the purpose of the study, the recording process, the time required, and the voluntary nature of answering the questions.

• Clarification of questions from the participant in advance.

• Collection of the code:

◦ The last two letters of your mother's maiden name

◦ The number of letters in your mother's (first) first name

◦ The last two letters of your father's (first) first name

◦ Your own birthday (only the day, not the month and/or year)

## 2. Experiencing pregnancy

• How did you feel when you found out you were pregnant?

• What were your circumstances when you found out you were pregnant?

• How did you experience the decision-making process?

## 3. Experience of the abortion

• How did you personally experience the abortion?

• Was there anything that helped you to process this procedure? (Protective factors)

If so, why?

• Was there anything that made it difficult for you to process this procedure? (Risk factors)

If so, why?

• Is there anything you would have liked to have had in order to be able to process the procedure (even) better?

If so, what and why?

## 4. Outcome

• How do you feel today about the abortion? (Positive/negative outcome)

• What thoughts and feelings do you have now when you think back on everything you experienced in connection with the abortion? (Positive/negative outcome)

• Does the topic still play a role in your everyday life? If so, to what extent? (Positive/negative outcome)

## 5. Concluding the conversation

• Is there anything else important about this topic that I forgot to ask?

• Clarify the emotional state of the participant, debrief if necessary, and refer again to the information sheet with the counseling services

• Thank the participant and say goodbye
